# Musical Emotions Recognition Using Entropy Features and Channel Optimization Based on EEG

**DOI:** 10.3390/e24121735

**Published:** 2022-11-28

**Authors:** Zun Xie, Jianwei Pan, Songjie Li, Jing Ren, Shao Qian, Ye Ye, Wei Bao

**Affiliations:** 1 Department of Arts and Design, Anhui University of Technology, Ma’anshan 243002, China; 2 Department of Management Science and Engineering, Anhui University of Technology, Ma’anshan 243002, China; 3 Department of Mechanical Engineering, Anhui University of Technology, Ma’anshan 243002, China

**Keywords:** EEG signals, musical emotions, emotion recognition, entropy, channel optimization

## Abstract

The dynamic of music is an important factor to arouse emotional experience, but current research mainly uses short-term artificial stimulus materials, which cannot effectively awaken complex emotions and reflect their dynamic brain response. In this paper, we used three long-term stimulus materials with many dynamic emotions inside: the “Waltz No. 2” containing pleasure and excitement, the “No. 14 Couplets” containing excitement, briskness, and nervousness, and the first movement of “Symphony No. 5 in C minor” containing passion, relaxation, cheerfulness, and nervousness. Approximate entropy (ApEn) and sample entropy (SampEn) were applied to extract the non-linear features of electroencephalogram (EEG) signals under long-term dynamic stimulation, and the K-Nearest Neighbor (KNN) method was used to recognize emotions. Further, a supervised feature vector dimensionality reduction method was proposed. Firstly, the optimal channel set for each subject was obtained by using a particle swarm optimization (PSO) algorithm, and then the number of times to select each channel in the optimal channel set of all subjects was counted. If the number was greater than or equal to the threshold, it was a common channel suitable for all subjects. The recognition results based on the optimal channel set demonstrated that each accuracy of two categories of emotions based on “Waltz No. 2” and three categories of emotions based on “No. 14 Couplets” was generally above 80%, respectively, and the recognition accuracy of four categories based on the first movement of “Symphony No. 5 in C minor” was about 70%. The recognition accuracy based on the common channel set was about 10% lower than that based on the optimal channel set, but not much different from that based on the whole channel set. This result suggested that the common channel could basically reflect the universal features of the whole subjects while realizing feature dimension reduction. The common channels were mainly distributed in the frontal lobe, central region, parietal lobe, occipital lobe, and temporal lobe. The channel number distributed in the frontal lobe was greater than the ones in other regions, indicating that the frontal lobe was the main emotional response region. Brain region topographic map based on the common channel set showed that there were differences in entropy intensity between different brain regions of the same emotion and the same brain region of different emotions. The number of times to select each channel in the optimal channel set of all 30 subjects showed that the principal component channels representing five brain regions were Fp1/F3 in the frontal lobe, CP5 in the central region, Pz in the parietal lobe, O2 in the occipital lobe, and T8 in the temporal lobe, respectively.

## 1. Introduction

Emotion is the psychological and physiological state of a human’s multiple feelings, thoughts and behaviors. It can reflect people’s psychological response to external stimuli and the accompanying physiological reactions. Emotions are produced in the cerebral cortex, and different emotions are the result of the synergistic effect of different cerebral cortical regions. In recent years, using EEG signals to study the emotion physiological mechanism and emotion recognition has become a hotspot [1,2,3,4]. The processes of emotion recognition mainly include emotion induction, EEG acquisition, feature extraction and emotion recognition.

As the root factor to arouse different emotions, the stimulus mode directly affects valence, arousal level and signal quality of EEG. At present, the main ways to arouse emotions are smell, text, picture, music [5,6], video [7,8], and virtual reality experience [9,10]. As the soul of music, emotion is expressed by the melody and rhythm of the music. Appreciating music is an emotional interaction between the author and the audience. The emotions in music may be conveyed to and resonate with the audience. This was a kind of emotional empathy induced by music and brought to the audience with the corresponding emotional experience [11,12]. Currently, music-related neurological research mainly focuses on exploring brain activity when some specific emotion is induced by music [13,14]. The research results showed that the asymmetry of EEG in the frontal lobe [15,16,17,18,19] is induced by different emotional valence; the left and right brain regions have different sensitivity to different types of music [20,21]; and the power changes of the brain in different bands are different during inducing musical emotions [22,23,24,25,26].

There are three types of EEG feature extraction of emotions: time domain, frequency domain and time-frequency domain [27,28,29,30,31]. Recently, non-linear dynamic features have also been gradually applied to feature extraction and analysis of emotional EEG [32]. Relevant indexes include Lyapunov exponent, correlation dimension, Lorenz scatter plot, Hurts exponent, and non-linear entropy. Compared non-linear feature extraction methods (e.g., fractal dimension, Lyapunov exponent, Hurst exponent, entropy) with feature extraction methods in the time domain, frequency domain, and time–frequency domain, it was found that non-linear analysis is very suitable for EEG signal-processing with a complex system [33]. In particular, the non-linear entropy has gained more and more attention in the feature extraction of EEG signals. The entropy describes the distribution probability of molecules of gaseous or fluid systems. Shannon first introduced the concept of information entropy based on thermodynamic entropy to describe the distribution of signal components. Up to now, many entropy algorithms have been proposed, mainly including ApEn [34,35], SampEn [36,37], Permutation entropy (PE) [38], Fuzzy entropy (FuzzyEn) [39], Shannon Wavelet entropy (SWE) [40], Hilbert-Huang spectral entropy (HHSE) [41], and multi-scale entropy (MSE) [42]. ApEn and SampEn are based on the time series, while above other methods are based on the frequency spectrum. ApEn statistics, however, lead to inconsistent results [34]. SampEn does not count templates as matching themselves and does not employ a template-wise strategy for calculating probabilities; therefore, SampEn can agree much better than ApEn statistics with theory, and can maintain relative consistency [36]. It has been proven that each algorithm has its advantages and limitations [43]. Identification accuracy is an important index to measure the performance of an algorithm, but it is necessary to comprehensively consider various evaluation indexes, such as robustness to noise, requirements for signal length and scale, and computational complexity, etc. The performance of the algorithm is closely related to the specific application object and the parameter selection.

Another crucial issue of EEG feature extraction is dimension reduction. Due to a large number of electrode channels in the EEG acquisition equipment, the redundant or less related to emotion EEG channel signals will affect the classification accuracy, and it will reduce the computational efficiency if all channel signals are involved in the classification operation. Therefore, channel optimization algorithms are quite necessary to be used. A deep neural network (DNN) was proposed for the channel selection and the classification of positive, neutral, and negative emotions, and the classification and recognition results based on four selected specific channels were better than the whole channels [44]. A novel group sparse canonical correlation analysis (GSCCA) method was proposed for channel selection and emotion analysis. The results of emotion recognition based on the SJTU emotion EEG dataset confirmed that the GSCCA method would outperform the state-of-the-art EEG-based emotion recognition approaches [45]. The 62 EEG channels were divided into five brain regions: frontal lobe, temporal lobe, central region, parietal lobe, and occipital lobe. The principal component analysis (PCA) method was used to only select the most important channels in each lobe-related channel, and the number of channels was reduced to five while retaining the main feature information [46]. 

As for the classification of musical emotions, the early research mainly used qualitative adjectives to construct discrete models and dimensional models to describe musical emotion tags. In 2008, a quantitative model of categorical emotions called Geneva emotional music scale (GEMS) was proposed [47]. The BRECVEM model was one of the most comprehensive models of musical emotion cognition, which elucidated the generation mechanism of musical emotion systematically [11]. Since explicit behaviors such as questionnaires, surveys, scoring, and clicking, etc. do not always reveal the subjects’ true emotions well, the psychophysiological characteristics such as blood pressure, pulse, electrocardiogram (ECG), skin electricity, eye electricity, EEG, etc., have attracted more and more attention. EEG technology can capture the related event potentials affected by timely emotions, and through the analysis of specific frequency bands, specific brain regions, and characteristic indexes, different emotions and the strength of emotions can be distinguished. The EEG-based methods for emotion classification usually adopt supervised and unsupervised learning methods in machine learning. Supervised learning methods mainly include neural networks (NNs), support vector machines (SVM) [48], KNN [49], extreme learning machine (ELM) [50], etc., and unsupervised learning methods commonly include K-Means clustering, fuzzy clustering, and self-organizing mapping [51]. Sohaib used KNN, Bayesian networks, SVM, artificial neural networks and regression trees to evaluate the performance of EEG emotion recognition, and the results confirmed that KNN and SVM had better recognition accuracy for small data sets [52]. In recent years, deep learning methods have been favored by more and more researchers, such as Convolutional Neural Network (CNN), Recurrent Neural Network (RNN), Generative Adversarial Network (GAN), Deep Belief Network (DBN), Artificial Neural Network (ANN), Long and Short Term Memory (LSTM), etc. These methods can be applied to classify more complex situations due to their advantages of the relatively shallow models in representational learning ability and high classification accuracy [53,54,55,56,57].

Traditional brain cognitive experiments are mostly based on short-term stimulus materials, and mark and classify the overall aroused emotions. For example, the Database for Emotion Analysis Using Physiological Signals (DEAP), which is widely used today, records 32 EEG signals of healthy subjects when they watch 40 different music videos with a duration of one minute and emotional assessment in the four dimensions of valence, arousal, dominance, and liking [58]. Due to the limited ability of short-term stimuli to induce emotions, and most short-term stimuli induce a single emotion, it cannot reflect the diversity and long-term dynamic variability of emotions, and cannot reflect the coherent perceptual process of the subject in a long period of time [59]. To stimulate the subjects to awaken emotional experiences similar to those in real life, long-term stimulus materials such as music, video, and movies have been used more and more frequently [60,61]. Furthermore, the research objectives of EEG’s emotion classification mainly focus on two classifications for two specific emotions and three classifications for positive [62,63], neutral, and negative emotions, and few examples of research involving four and more multi-emotional classifications have been explored [64,65]. The current sample data mainly takes the emotions aroused by music as whole to mark. There is a lack of classification research based on the fragment marked and a lack of classification research about emotion change in the same music material. 

In this paper, to obtain continuous emotional experience and corresponding EEG sample data under dynamic music stimulation, long-term stimulus materials containing two or more emotions were used to induce subjects to produce diverse and long-term dynamic emotions. To obtain the specific neurological features of different emotional experiences, the PSO algorithm taking the emotion recognition accuracy as the objective function was used to select the optimal channels for each subject. Further, a method to construct the common channel set was proposed, which can basically reflect the universal features of the whole subjects while realizing feature dimension reduction.

## 2. Materials and Methods

### 2.1. EEG Experiment

#### 2.1.1. Materials

Musicians think that the combination of symphony and performance video can make the music more emotional. The consensus is that the audiences’ emotional experience of the music is more extreme (stronger or weaker) when visual information is added to the music. The audience has a stronger emotional experience when watching the live video of the symphony orchestra than just listening to the music [66]. The combination of live shows and music can achieve a better performance in cognition tasks [67]. Based on these studies, three music videos of the live concert version are used as the experimental materials to arouse the corresponding emotions of subjects. There are a variety of emotional changes in the three experimental materials, corresponding to the two, three, and four classifications, respectively. Table 1 describes three music materials used in experiments (all the music experimental materials can be obtained from the corresponding author). The music material was edited in advance. The time segments of each music material are marked with corresponding emotions according to five music professionals’ suggestions. Two main emotions are contained in “Waltz No. 2” (composed by Dmitri Shostakovich), three main emotions are contained in “No. 14 Couplets” (i.e., Toreador Song, composed by Georges Bizet), and four main emotions are contained in the first movement of the “Symphony No. 5 in C minor” (composed by Beethoven). The time segments corresponding to various emotions and sample statistical information are shown in Table 1.

#### 2.1.2. EEG Signal Acquisition and Sample Data

Data were obtained from 30 subjects (13 men, aged 18–25, right-handed). All subjects reported normal or corrected vision, normal hearing, and no history of neurological disease. Subjects had no formal musical training and had never learned to play a musical instrument. The experiments were approved by the Ethical Review Committee of the Biomedical Research Ethics Committee of Anhui University of Technology (approval date: 14 October 2021), and all subjects signed informed consent. All subjects were numbered sequentially and randomly divided into three groups with ten subjects in each group (Table 1). The experiment was completed in a closed room with constant temperature and isolation from noise interference. The subjects sat alone in front of the computer monitor. Music was played through an external stereo, and the volume was adjusted to the appropriate decibel by the subject before the experiment. Each subject was required to watch and listen to one music material (music videos of the live concert version). Subjects click “Yes” according to the computer interface if ready, and then, the computer displays “Start”, and after ten seconds, the music material corresponding to the specified subjects was played. After the music was played, the subject was asked to rate the emotional arousal and potency of each segment. All stimulus presentations and marks were synchronized with EEG signals through E-prime 3.0. During the experiment, subjects were required to keep their bodies stable to reduce the interference of EMG.

EEG signals were obtained using 32-channel ActiChamp (use BP-09100 as the base module and BP-09110 as a 32-channel module) at 500 Hz sampling frequency (electrodes positioned according to the International 10–20 Electrode Placement System). The BrainVision Recorder was used to configure channel parameters and record EEG signals. The electrode impedance at each site is below 10 kΩ. The signal was referenced against Fz, and later re-referenced against TP9 and TP10 on bilateral papillary. BrainVision Analyzer was used for data pre-processing. The notch filter was applied to the data for removing the 50 Hz frequency of the power supply, and a first-order low-pass Butterworth filter with a frequency of 0.5 to 47 Hz was applied to the data. Ocular corrections were conducted using independent component analysis (ICA). 

After signal preprocessing, according to the results of emotional arousal rating and data preprocessing, EEG data of 30 subjects are available. The emotion signals were divided into series samples with one-second intervals (500 EEG sampling points per second). The sample size of each emotion is listed in Table 1. There are 114 pieces of pleasant sample data and 88 pieces of excited sample data in “No. 2 Waltz”, 71 pieces of sample data corresponding to excitement, 59 pieces of sample data corresponding to briskness, and 10 pieces of sample data corresponding to nervousness in “No. 14 Couplets”. In the first movement of “Symphony No. 5 in C minor”, there are 48 pieces of sample data corresponding to passion, 59 pieces of sample data corresponding to relaxation, 36 pieces of sample data corresponding to cheerfulness, and 97 pieces of sample data corresponding to nervousness.

### 2.2. Feature Extraction of EEG Signals

Feature extraction is to highlight the representative characteristics of some modes by using a method, such as EEG sequence signals. Since entropy was proven to be an effective method to get information from EEG [68] and EEG entropy features can be used as an important index for emotion classification [69,70,71]. ApEn, SampEn, permutation entropy, and wavelet exotic entropy were used as characteristic values for classification, and the results showed that using the joint features of ApEn and SampEn could obtain better performance. SampEn is an improved index based on ApEn with better consistency. The calculation methods of the two indexes are as follows.

#### 2.2.1. Approximate Entropy

The calculation steps of ApEn are [34]:

Step 1: Assume time sequence vector *X* as an *N* (*N* = 500) sequence set of original signal {x(1),x(2),…,x(N)}, and reconstruct the *i*th element in sequence to be *m*-dimensional vector $x_{m}(i)$, $x_{m}(i)=\{x(i),x(i+1),\ldots ,x(i+m-1)\}$, where 1≤i≤N−m+1. Then, calculate the similarity distance $d(X_{m}(i),X_{m}(j))$ of any two vectors $X_{m}(i)$, $X_{m}(j)$ according to Equation (1).

(1) $$d(X_{m}(i),X_{m}(j))=\max|x(i+k)-x(j+k)|$$

where i,j=1,2,…,N−m+1, k=0,1,…,m−1.

Step 2: Set parameter r as the similarity tolerance, count the number of distances that satisfies the inequality $d(X_{m}(i),X_{m}(j))<r$, and calculate the ratio of this number to N−m+1. The ratio $C_{i}^{m}(r)$ is defined as follows.

(2) $$C_{i}^{m}(r)=\frac{1}{N-m+1}\mathrm{num}\{d(X_{m}(i),X_{m}(j))<r\}$$

Step 3: Calculate the logarithm mean of all $C_{i}^{m}(r)$, and the obtained result is denoted as $\phi^{m}(r)$.

(3) $$\varphi^{m}(r)=\frac{1}{N-m+1}\sum_{i=1}^{N-m+1}{\mathrm{lnC}}_{i}^{m}(r)$$

Step 4: Increase the dimension m to m+1, repeat the above steps 1–3 to get $\phi^{m+1}(r)$. The value of ApEn is calculated by Equation (4).

(4) $$\mathrm{ApEn}=\phi^{m}(r)-\phi^{m+1}(r)$$

#### 2.2.2. Sample Entropy

The calculation steps of SampEn are [36]:

Step 1 and Step 2 are the same as calculating ApEn, but different with $B_{i}^{m}(r)$. Let replace $C_{i}^{m}(r)$ by Equation (5).

(5) $$B_{i}^{m}(r)=\frac{1}{N-m}\mathrm{num}\{d(X_{m}(i),X_{m}(j))<r,j\neq i\}$$

Step 3: Calculate the mean of all $B_{i}^{m}(r)$, and the result is denoted as $A^{m}(r)$.

(6) $$A^{m}(r)=\frac{1}{N-m+1}\sum_{i=1}^{N-m+1}B_{i}^{m}(r)$$

Step 4: Increase the dimension m to m+1, repeat above steps 1–3, and to get $A^{m+1}(r)$. The formula of the SampEn is:

(7) $$\mathrm{SampEn}=\mathrm{Ln}A^{m}(r)-\mathrm{Ln}A^{m+1}(r)$$

In this paper, parameter values are *m =* 2, the similarity tolerance r=0.15STD, where STD is the standard deviation of the time series, and $\mathrm{STD}=\sqrt{\frac{1}{N}\overset{N}{\underset{i=1}{\sum }}[x\left(i\right)-\frac{1}{N}\overset{N}{\underset{i=1}{\sum }}x\left(i\right)}{]}^{2}$.

### 2.3. KNN Classification Algorithm

In the process of emotion recognition, the goal is to extract the features of EEG signals and recognize various emotions by using appropriate algorithms. At present, those algorithms, such as decision trees, KNN, SVM, and neural networks, have been widely used for the classification of emotional EEG. In our previous study, KNN, SVM, and ELM were used to classify and identify the emotions, and the KNN algorithm achieved the best performance, so KNN was finally selected in this paper.

The core idea of KNN is “birds of a feather flock together “. The algorithm principle is: given a training sample with known classification, we calculate the distance between the test sample and all the training samples. Then, we find out the *K* training samples closest to the test samples and take the category with the largest proportion of *K* training samples as the category of the test samples. Here, the functions of measuring distance are Euclidean distance, Manhattan distance, and Heming distance. In this paper, the Euclidean distance is selected, and the parameter *K* is 2. The detailed process of the KNN algorithm can be found in the reference [46]. The recognition accuracy is defined as the ratio of the number of correct samples identified by a classifier of the total number of samples in the test set. 

### 2.4. Channel Selection Based on the PSO Algorithm

In this paper, each data acquisition channel corresponds to one electrode. The PSO algorithm is used to select the optimal channels of the EEG signal for decreasing the number of data dimensions. The calculation steps are (Pseudo-code of the PSO algorithm shown in Algorithm 1):

Step 1: Let set the population size of particles (i.e., feasible solutions) to be *n* and the maximum number of iterations $t_{\max}$, and randomly initialize the position $X_{i}=(X_{i1},X_{i2},\ldots ,X_{iD})$, $X\in [-X_{\max},X_{\max}]$ and the velocity of position change $V_{i}=(V_{i1},V_{i2},\ldots ,V_{iD})$, $V\in [-V_{\max},V_{\max}]$(X∈[−10,10], V∈[−4,4]) of the particle i (i=1,2,…,n) in *D*-dimension search space, where *D* is the number of channels 30, the population size *n* is 50, and the maximum number of iterations *t*_max_ is 100.

Step 2: The fitness function is defined with the recognition accuracy, and it is calculated as follows:

(1) For the position vector of the particle i, we use the Sigmoid function $S(x)=1/\left(1+e^{-x}\right)$ to linearly map the position vector, and obtain the weight of 30 channels in the range [0, 1], and then set the threshold to be 0.5. If some channel’s weight is greater than 0.5, the channel is selected, and the value is set to be 1 compulsorily, otherwise, the channel is abandoned, and the value is set to be 0 compulsorily.
(2) For the selected channels, we use the KNN algorithm in Section 2.3 to calculate the fitness value of the *i*th particle based on the eigenvalue calculation in Section 2.2. During calculating the fitness value, the ten-fold cross-validation method is used to randomly divide the two eigenvalues (ApEn and SampEn) into 10 parts. Each time, one part is selected as the test set, and the other 9 parts are used as the training set, then, KNN is applied to obtain the corresponding recognition accuracy. After running 10 times in turn, we take the average of 10 runs as the fitness value.
(3) Repeat the above step (1) and (2) for each particle to obtain the fitness value of all particles. $P_{i}^{k}=(P_{i1}^{k},P_{i2}^{k},\ldots ,P_{iD}^{k})$ is defined as the position vector corresponding to the optimal fitness value of the *i*th particle in the *t* iterative process, $P_{g}^{k}=(P_{g1}^{k},P_{g2}^{k},\ldots ,P_{gD}^{k})$ is defined as the position vector corresponding to the global optimal solution (that is, the maximum fitness value of the population) in the *t* iterative process, where t∈[0,k], k is the number of current iterations.

Step 3: Let us update the velocity and position of all particles (i=1,2,…,n). Then the updating formulas are:

(8) $$V_{ij}^{k+1}=\lambda V_{ij}^{k}+c_{1}r_{1}(P_{ij}^{k}-X_{ij}^{k})+c_{2}r_{2}(P_{gj}^{k}-X_{ij}^{k}),\;j\in (1,2,\ldots ,D)$$

(9) $$X_{ij}^{k+1}=X_{ij}^{k}+V_{ij}^{k+1},\;j\in (1,2,\ldots ,D)$$

The right side of Equation (8) consists of three parts in order: “inertia”, “cognition”, and “society” [72]. The “inertia” makes the particles maintain their original speed. The “cognition” makes individuals tend to be the historically best locations. The “society” reflects the cooperation and sharing the information among particles, which makes particles be close to the best location of the population. λ is inertia weight, $c_{1}$ and $c_{2}$ are learning factors, $r_{1}$ and $r_{2}$ are uniform random numbers range in (0,1). Here, we set learning factors $c_{1}=c_{2}=1.49445$ and inertia weight λ=1. If $V>V_{\max}$ or $V<-V_{\max}$ after updating the particle’s velocity, we set $V=V_{\max}$ or $V=-V_{\max}$. If the particle’s position exceeds the upper and lower limits during the update process, the processing method is the same as the velocity.

Step 4: If the maximum number of iterations is reached or the convergence condition is met, the process ends. Otherwise, the above steps 2 to 3 are repeated.
**Algorithm 1.** Pseudo-code of the PSO algorithm.Input: the maximum number of iterations *t*_max_, total population size *n,* dimension D. Output: optimal channel number, best fitness.1.      Set the parameters and generate the initial population randomly. 2.      Calculate the fitness value of the population.         For    *i* = 1→*n*
                  For    *j* = 1→D                                   If $1/\left(1+e^{-X_{ij}}\right)>0.5$
                                  *j* is selected, perform feature extraction of EEG signals for channel *j*. Calculate ApEn and SampEn of all the sample data by Equations (1)–(7).                                   End If                   End                   For the selected channels, the sample data is divided into 10 parts randomly, where one part is selected as the test set, and the remaining nine parts are used as the training set. Then take the average of 10 times as the fitness value by KNN.         End 3.    Update position vectors $p_{j}^{k}$, $p_{g}^{k}$. 4.    For    *t* = 1→*t*_max_
5.                Use Equations (8)–(9) to update the position of the population. 6.                Repeat 2 and 3. 7.                Determine whether the maximum iterations is reached, and if so, the iteration ends, and the optimal solution is output. Otherwise, the cycle continues. 8.    End

## 3. Results

### 3.1. Two Classifications of Emotions Based on “Waltz No. 2”

Table 2 presents the optimal channel selection of ten subjects by the PSO algorithm based on “Waltz No. 2”. Label “1” means to select this channel, and “0” means not to select this channel. The last column counts the total number of times that each subject selects this channel. The last row is the recognition accuracy of each subject using the optimal channels.

**Figure 1:**
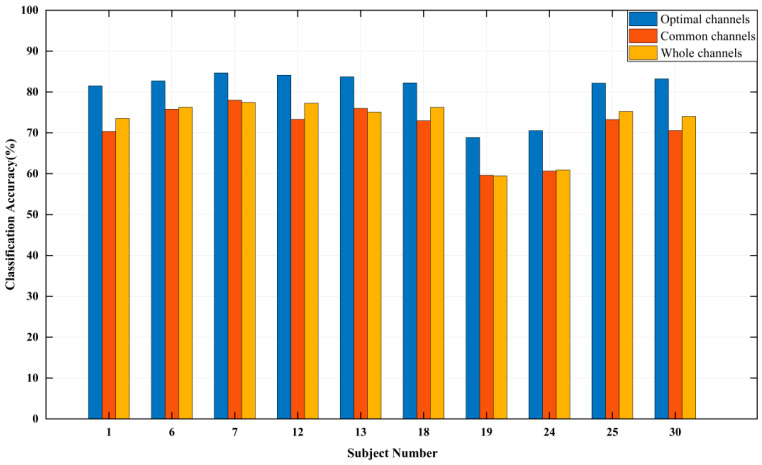
Emotion classification accuracy of “Waltz No. 2“.

It can be seen from Table 2, the recognition accuracy of subjects is more than 80% except subjects No. 19 and No. 24. For different subjects, the names and number of optimal channels are different (the number of channels is in the 14–18 range), and there are significant personalized differences. Therefore, it is necessary to find out the common suitable channels for all subjects. The common channel set with the total number of selected times of six or more is: {F3, F7, CP5, CP1, Pz, P3, P7, O1, O2, CP2, T8, FC2, F8}, 13 channels in total. The classification and recognition accuracy of 10 subjects based on the common channels, the whole channels, and the optimal channels are shown in Figure 1. Figure 1 presents that compared with the whole channels and the optimal channels, the difference in recognition accuracy using the common channels is −3.96% to 0.93%, and −12.65% to −6.68%, respectively. In summary, the common channels can not only realize feature dimension reduction, but also basically reflect the common characteristics of all subjects.

**Figure 2:**
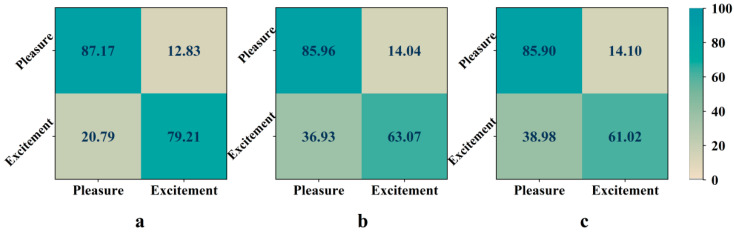
Emotional confusion matrix of subject No. 13 based on “Waltz No. 2”. (**a**) Optimal channels. (**b**) Common channels. (**c**) Whole channels. The value in the matrix denotes the percentage of emotion recognition result.

Figure 2 illustrates the confusion matrix of the emotion recognition result of subject No. 13. The row labels represent real emotions, and the column labels represent recognized emotions. The value in the matrix is the ratio of the sample size of the output emotion category to the sample size of real emotion. It can be observed from Figure 2 that the recognition accuracy of pleasure is high, while excitement is very difficult to be distinguished. Compared with the common channels and the whole channels, the overall recognition accuracy of optimal channels is low, and the reason is that the probability of the excitement mood being identified mistakenly as pleasure increases.

**Figure 3:**
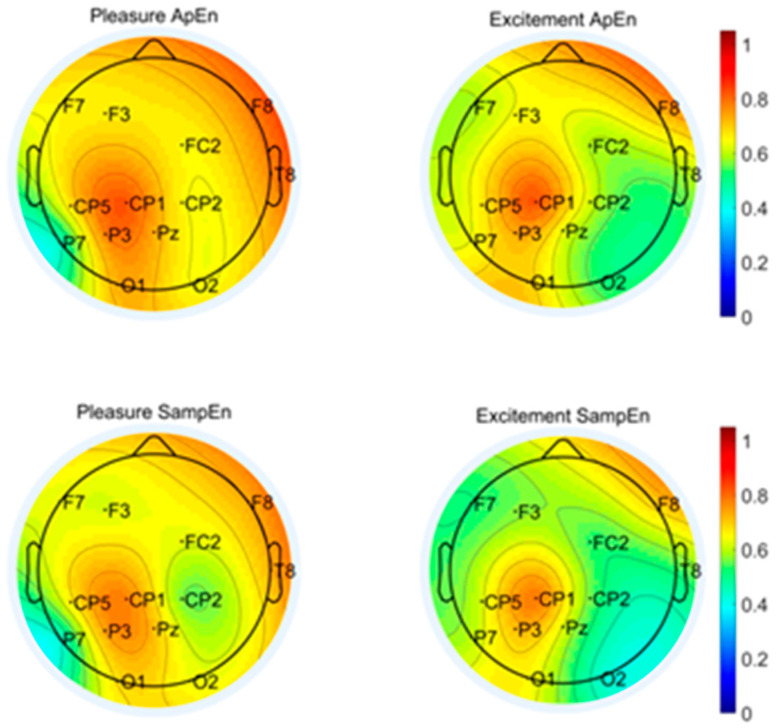
Brain region topographic map of “No. 2 Waltz” based on the common channel set.

To observe the difference between the emotions of pleasure and excitement in the common channels based on “Waltz No. 2”, Figure 3 presents the brain region topographic map of the average value of ten subjects’ ApEn and SampEn. The brain region distribution of ApEn and SampEn are mostly the same, and the intensity of the ApEn is slightly higher than the SampEn. The reason may be that the irregularity of EEG signals has a more significant influence on ApEn than that on SampEn. It also can be found that 13 common channels are distributed in five brain regions. There are F3/F7/FC2/F8 in the frontal lobe, CP5/CP1/CP2 in the central region, Pz/P3/P7 in the parietal lobe, O1/O2 in the occipital lobe, and T8 in the temporal lobe. There are certain differences in the entropy values between the same brain regions with different emotions and between different brain regions with the same emotions.

The overall entropy value of the other common channels is higher for pleasure except P7 in the left parietal. For the excitement mood, the brain regions with higher entropy values occur near CP1 in the central region and F8 in the right frontal lobe, while the entropy values of the other brain regions are relatively low. The response of the EEG entropy of pleasure in the right temporal lobe T8 is significantly stronger than that of the excitement mood, while the response of the EEG entropy of excitement in the left parietal lobe P7 is significantly stronger than that of pleasure.

### 3.2. Three Classification of Emotions Based on “No. 14 Couplets”

**Figure 4:**
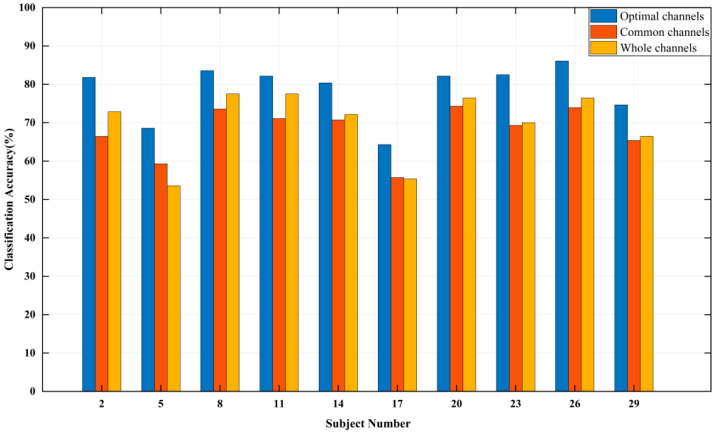
The emotion classification accuracy of “No. 14 Couplets”.

Table 3 presents the results of optimal channels selection and the recognition accuracy based on “No.14 Couplets”. The recognition accuracy of subjects is all higher than 80% except subjects No.5, 17 and 29. For different subjects, the number of optimal channels is between 13 and 20. The set of common channels with the total number of selection times of six or more is: {Fp1, F3, FT9, FC5, FC1, C3, CP5, P3, O1, Oz, O2, P8, CP6, CP2, T8, F4}, 16 channels in total. Figure 4 illustrates the classification accuracy of ten subjects based on the common channels, the whole channels, and the optimal channels. The results indicate that compared with the whole channels, the difference of recognition accuracy of the common channels is −6.43% to 5.72%, and compared with the optimal channels, this difference is −15.36% to −7.85%.

**Figure 5:**
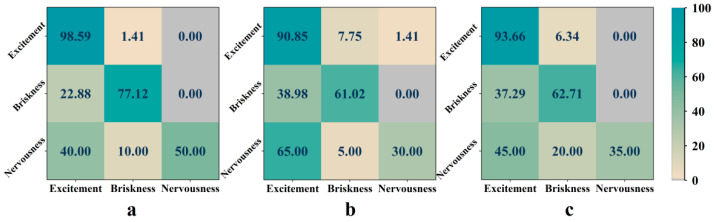
Emotional confusion matrix of subject No. 26 based on “No. 14 Couplets”. (**a**) Optimal channels. (**b**) Common channels. (**c**) Whole channels. The value in the matrix denotes the percentage of emotion recognition result.

Figure 5 represents the confusion matrix of the emotion recognition result of subject No. 26. The recognition accuracy of excitement is the highest, and the recognition accuracy of briskness is medium with a high probability of being recognized as excitement. It is the most difficult to identify nervousness, while easy to be identified as excitement. The reasons for a low recognition accuracy of nervousness are: (1) There are only 10 pieces of sample data correlating to nervousness, and the number of samples is obviously unbalanced compared with the other two emotions. This imbalance makes it difficult to be recognized. (2) The music segment correlating to nervousness is too short (10 s). This may make the subjects have less time to complete the emotion transformation, or to be directly dominated by the emotions of the next segment when they experience the present short segment. Therefore, in the 10 s, maybe the actual emotional experience of subjects is not nervousness, while it is incorrectly labeled as nervousness. This leads to the inconsistency of emotion marked beforehand with emotion recognized based on the EEG signals.

**Figure 6:**
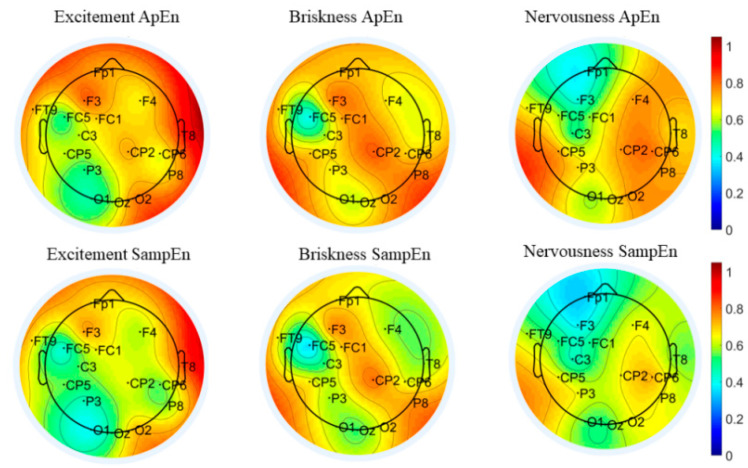
Brain region topographic map of “No. 14 Couplets” based on common channel set.

Figure 6 represents the topographic map of the brain region characteristics of 10 subjects based on “No. 14 Couplets”. It can be noticed that 16 common channels are distributed in five brain regions, and mainly concentrated in the frontal lobe and central region, a little more on the left side. Here, Fp1/F3/FT9/FC5/FC1/F4 are distributed in the frontal lobe, C3/CP5/CP6/CP2 in the central region, P3/P8 in the parietal lobe, and O1/Oz/O2 in the occipital lobe, T8 in the temporal lobe. The response of the EEG entropy of excitement in the right temporal T8 is significantly stronger than that of briskness and nervousness. The EEG entropy of briskness in the left prefrontal Fp1 is suppressed, and the intensity is significantly weaker than that of excitement and briskness. The response of the EEG entropy of briskness in the central region CP2 is slightly stronger than that of excitement and nervousness, while the response of the EEG entropy of FC5 in the left frontal is slightly weaker than that of excitement and nervousness.

### 3.3. Four Emotions Classifications Based on “Symphony No. 5 in C Minor”

**Figure 7:**
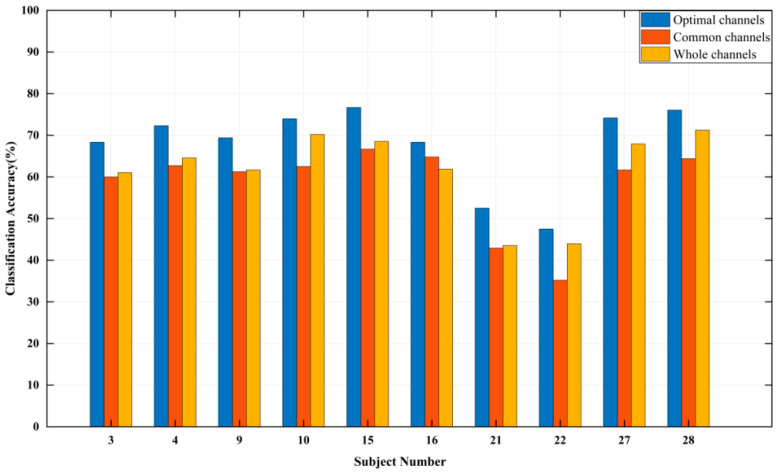
The classification accuracy of “Symphony No. 5 in C minor”.

As can be seen from Table 4, the recognition accuracy of subjects is all around 70% except subjects No. 21 and No. 22. The recognition accuracy of four classifications is lower than the ones of the two classifications (Table 2) and three classifications (Table 3). For different subjects, the number of optimal channels is in the 12–21 range. The set of common channels with the total number of selection times of six or more is: {Fp1, F3, F7, FT9, FC1, CP5, Pz, P3, O1, Oz, O2, P4, P8, CP2, T8, FC2, Fp2, Fz}, 18 channels in total. The recognition accuracy of 10 subjects based on the common channels, the whole channels and the optimal channels are shown in Figure 7. The results indicate that compared with the whole channels, the difference of recognition accuracy using the common channels is −8.75% to 2.91%, and compared with the optimal channels, this difference is −14.58% to −3.54%.

**Figure 8:**
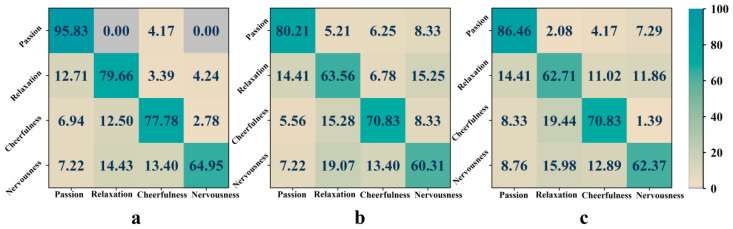
Emotional confusion matrix of subject No. 15 based on “Symphony No. 5 in C minor”. (**a**) Optimal channels. (**b**) Common channels. (**c**) Whole channels. The value in the matrix denotes the percentage of emotion recognition result.

The emotional confusion matrix for the recognition result of subject No. 15 is given in Figure 8. The recognition accuracy of passion is the highest, nervousness is the lowest, and nervousness is very easy to be misidentified as cheerfulness or relaxation. Comparing the optimal channels with the common channels and the whole channels, the difference is mainly reflected in the recognition accuracy of passion and relaxation, and the probability of being wrongly identified as nervousness increases. Generally speaking, the recognition accuracy of positive emotions is high, and negative emotions are more difficult to identify. Meanwhile, nervousness has certain negative characteristics compared with the other three emotions, so its recognition accuracy is low and easy to be confused.

**Figure 9:**
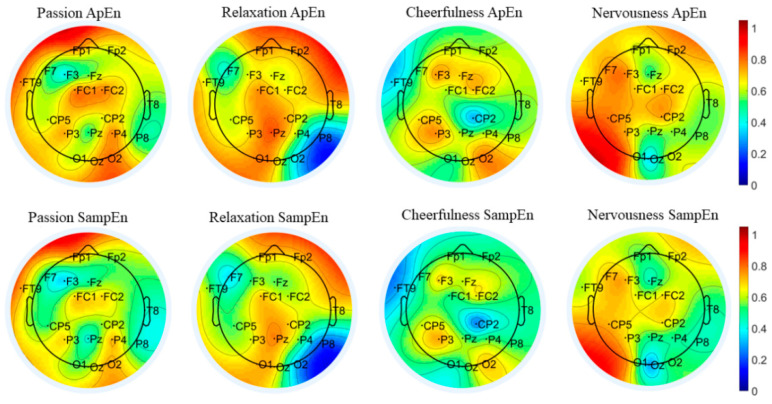
Brain region topographic map of “Symphony No.5 in C minor” based on the common channel set.

Figure 9 presents the characteristic topographic map of the brain regions of 10 subjects based on the first movement of “Symphony No.5 in C minor”. We observe that 18 common channels are distributed in five brain regions, mainly concentrated in the frontal and parietal regions. There are Fp1/F3/F7/FT9/FC1/FC2/Fp2/Fz in the frontal lobe, CP5/CP2 in the central region, Pz/P3/P4/P8 in the parietal lobe, O1/Oz/O2 in the occipital lobe, and T8 in the temporal lobe. The response of the EEG entropy of passion in the left prefrontal region Fp1 is significantly stronger than that of the other three emotions, while the entropy of F3 in the left frontal is weaker than the other three emotions. The response of the EEG entropy of relaxation in the vicinity of P8 in the right parietal is significantly suppressed. The entropy value in the vicinity of Fp2 in the right frontal lobe to the T8 channel in the temporal lobe is significantly higher than that of the other three emotions. The entropy of the cheerfulness in whole brain regions is weak, especially in the central region CP2 and in the left frontal FT9. The EEG entropy of nervousness in the region near P3 of the left parietal is significantly stronger than that of the other three emotions.

## 4. Discussion

The current EEG-based research on musical emotions mainly adopts short-term stimulus materials. However, the ability of short-term stimulation to induce emotion is limited and single, which cannot reflect the corresponding brain response under complex dynamic emotional changes. The dynamic nature is one of the reasons why music can stimulate people’s strong emotional experience; however, short-term stimulation often does not have long-term dynamic characteristics. Therefore, three long-term stimulus materials are adopted in this paper. The EEG responses and classification results based on “Waltz No. 2” (including the dynamic changes of two emotions), “No. 14 Couplets” (including the dynamic changes of three emotions), and “Symphony No. 5 in C minor” (including the dynamic changes of four emotions) all show the diversity and dynamic emotional experience of the subjects.

The emotional cognitive process and EEG response under long-term music stimulation have strong non-linear characteristics, and entropy is an important index to describe the complexity of this system. Based on ApEn and SampEn with common channels, the brain region topographic maps of the overall average entropy value of the subjects are depicted, and the results suggest that there are differences in the distribution of the entropy intensity between different emotions. Those distribution differences may be the foundation for emotional classification and identification. Murugappan et al. [73] proposed that EEG entropy can be used as an effective index of emotion classification. Their research showed that EEG entropy in the emotional state is smaller than that in the non-emotional state, and the accuracy of emotion recognition based on entropy is higher than that in the time domain.

Selecting optimal channels and constructing the common channel set are important steps to reduce the dimension of data. Meanwhile, using an optimal channel set can improve the accuracy of emotion recognition for a single subject, and the recognition accuracy based on the common channel set is lower than that based on the optimal channel set, but not much different from that based on the whole channel set. At present, there are many methods for a feature or channel selection including linear discriminant analysis (LDA), principal components analysis (PCA), singular value decomposition (SVD), QR decomposition with column pivoting (QRP), etc. These methods belong to unsupervised methods, which do not use category label information. In this paper, the PSO algorithm was used to select channels. First, the optimal channel set for each subject was obtained, and then a threshold value was determined by counting the selection times of each channel corresponding to the optimal channel set of all subjects (it takes six times, that is, the channel appears in the optimal channel set of six subjects out of ten subjects). If the selection times of a certain channel are greater than or equal to the threshold, it is a common channel suitable for all subjects. The method in this paper belongs to the supervised eigenvector dimensionality reduction method because the recognition rate should be taken as the objective function in channel optimization selection.

Based on the optimal channel set, for the emotional two classifications in “Waltz No. 2” and the emotional three classifications in “No. 14 Couplets”, the accuracies of emotion recognition are both more than 80% for 70% of the subjects. The same methods were applied to the emotional four classifications based on the first movement of “Symphony No. 5 in C minor”, and the recognition accuracy is about 70% for 80% of the subjects. Subsequently, corresponding to each experimental group, the common channel set was constructed based on the optimal channel set of all subjects. Based on the common channel set, the average recognition accuracies for the emotional two classifications in “Waltz No. 2” and the emotional three classifications in “No. 14 Couplets” are both about 70%. For the emotional four classifications in the first movement of “Symphony No. 5 in C minor”, the average recognition accuracy is about 60%. The diversity and dynamics of emotions increase the difficulty of recognition. At present, there are few research results on the four and above categories of emotions [74,75,76], and the classification samples are all based on the overall labels of short-term music fragments. For the research on emotion classification with the mode of long-term stimulation, Kaur [63] studied the emotion classification of calm, anger, and happiness based on video evoked EEG signal, and used SVM to obtain an average accuracy of 60%. Liu [64] proposed an emotion recognition system based on EEG, in which emotion is induced by a real-time movie. The average recognition accuracy of positive emotions and negative emotions reaches 86.63%, the recognition accuracy of three positive emotions (joy, entertainment, gentleness) reaches 86.43%, and the recognition accuracy of four negative emotions (anger, disgust, fear, sadness) is about 65.09%. The recognition accuracy is slightly greater than the results of two, three, and four classifications in this paper. The reason may be that the real-time movie has a long period of time (meaning a large amount of sample data and good balance), and the stimulation of a story plot and visuals are stronger than the music.

Compared with the whole channel set, the difference in emotion recognition accuracy using the common channel set is about −8% to 6%. Therefore, the common channels can not only realize feature dimension reduction, but also basically reflect the whole universal characteristics of the subjects. In the aspect of channel distribution in brain regions, we find that the common channels are distributed in five brain regions for all three group subjects; in addition, the number of channels distributed in the frontal lobe is more than that in the other four brain regions, accounting for 4/13, 6/16, and 8/18, respectively. This result indicates that the frontal lobe region is the main brain region responding to musical emotions. Furthermore, based on the statistics of the optimal channel set of 30 subjects, the total frequency of the optimal channel selection is 498, and then the average frequency of the channel selected as the optimal channel for any of the 30 channels is 16.6 (498/30). The frequency ratio of selecting optimal channel (abbreviated as FRSOC in this paper) is defined as: the ratio of the total frequency of this channel selected as the optimal channel by 30 subjects to the average frequency. This index reflects the relative strength of the channel selected as the optimal channel. Meanwhile, the optimal channel selection rate (abbreviated as OCSR in this paper) is defined as: the ratio of the number of subjects selecting the channel as the optimal channel to 30 subjects. This index reflects the breadth of the channel selected as the optimal channel.

According to the above two indexes of each channel (shown in Table 5), it can be seen that the FRSOC of six channels (namely) are 1.265 for Fp1, F3, Pz, and O2 and 1.325 for CP5 and T8, respectively, with the corresponding OCSR ≥ 70%, indicating that compared with other channels, these six channels have advantages in term of the strength and breadth, so the above six channels are considered as the principal component channels of the EEG response in the three experiments, which are mainly distributed in five brain regions, including Fp1/F3 in the frontal lobe, CP5 in the central region, Pz in the partial lobe, O2 in the occipital lobe, and T8 in the temporal lobe. The FRSOC of three channels, namely C4, FT10, and FC6, are 0.663 with the corresponding OCSR = 36.7%, indicating that these three channels have no advantages in terms of the strength and breadth, so they are considered as weak-related channels of EEG response in the three experiments, which are mainly distributed in the right frontal lobe and the right central region.

## 5. Conclusions

In this paper, EEG feature extraction (based on ApEn and SampEn), classification, and recognition (KNN used) were explored for the two, three, and four classifications, respectively, for the emotions aroused by the above three music materials.

Compared with short-term artificial stimulation, long-term stimulation may have completely different effects on the sensory processing of music attributes, the perception and understanding of the music’s meaning, and the awakening and imagination of individual emotional consciousness. To further improve the dynamics and immersion (or arousal) of subjects’ emotional experience, it is suggested that VR can be used as an emotional stimulus in future musical emotion research [77]. Entropy has advantages and characteristics in depicting the dynamic and non-linear changes of complex systems. It would be interesting to explore the time dynamics, clusters of stable emotion periods, and critical points of change based on different entropy features. It is also necessary to further mine the non-linear features of EEG signals based on entropy, such as WT-CompEn [78], for more comprehensive and accurate feature extraction.

To improve recognition accuracy and calculation efficiency, the PSO algorithm was used to select the optimal channels of EEG signals. Furthermore, in each group of experiments, an overall set of common channels for all participants was constructed, and the brain region response to music is analyzed based on the optimal channel set to obtain the universal characteristics of all participants.

## Figures and Tables

**Table 1:** Experimental materials and sample statistical information.

Material	Sample Information			Emotion (Sample Size)	Subject
	Time Segment	Emotion Aroused	Sample Size		
Waltz No. 2	0:20–1:16	Pleasure	57	Pleasure (114) Excitement (88)	No. 1, 6, 7, 12, 13, 18, 19, 24, 25, 30
	1:17–2:21	Excitement	65		
	2:22–3:01	Pleasure	40		
	3:02–3:24	Excitement	23		
	3:25–3:41	Pleasure	17		
No. 14 Couplets	0:06–0:33	Excitement	28	Briskness (59) Excitement (71) Nervousness (10)	No. 2, 5, 8, 11, 14, 17, 20, 23, 26, 29
	0:34–0:42	Briskness	9		
	0:43–0:52	Nervousness	10		
	0:53–1:07	Excitement	15		
	1:08–1:57	Briskness	50		
	1:58–2:25	Excitement	28		
The first movement of “Symphony No. 5 in C minor”	0:16–0:45	Passion	30	Passion (48) Relaxation (59) Cheerfulness (36) Nervousness (97)	No. 3, 4, 9, 10, 15, 16, 21, 22, 27, 28
	0:46–1:05	Relaxation	20		
	1:06–1:23	Cheerfulness	18		
	1:24–1:41	Passion	18		
	1:42–1:53	Relaxation	12		
	1:54–2:10	Nervousness	17		
	2:11–2:37	Relaxation	27		
	2:38–2:55	Cheerfulness	18		
	2:56–4:15	Nervousness	80		

**Table 2:** Optimal channels selection and recognition accuracy of “Waltz No. 2”.

Channel	Subject										Total
	1	6	7	12	13	18	19	24	25	30	
Fp1	0	1	1	1	0	0	1	1	0	0	5
F3	1	1	0	1	1	1	1	1	1	0	8
F7	1	0	1	1	1	0	0	1	1	0	6
FT9	1	0	1	0	0	1	1	0	0	1	5
FC5	0	0	1	0	0	0	0	0	1	1	3
FC1	1	0	0	1	0	1	0	0	1	0	4
C3	0	0	0	0	0	0	0	0	0	1	1
T7	1	1	1	1	0	0	1	0	0	0	5
CP5	0	1	1	0	1	1	1	0	1	1	7
CP1	1	0	1	1	1	0	0	0	1	1	6
Pz	1	1	1	1	1	1	1	1	1	1	10
P3	0	1	1	0	0	1	1	1	1	0	6
P7	1	0	1	1	1	1	0	0	1	1	7
O1	0	0	1	1	1	1	0	1	1	1	7
Oz	1	0	0	0	1	0	1	0	1	0	4
O2	1	0	1	1	0	0	1	1	1	0	6
P4	0	1	1	0	0	1	0	1	0	0	4
P8	1	1	1	1	0	0	0	1	0	0	5
CP6	1	1	0	0	0	1	0	0	1	1	5
CP2	1	1	1	1	1	1	0	1	0	0	7
Cz	0	1	1	1	0	0	1	1	0	0	5
C4	0	0	0	1	0	0	1	1	0	0	3
T8	1	0	1	1	1	1	1	1	0	1	8
FT10	1	0	0	1	1	1	0	0	0	0	4
FC6	0	0	0	1	0	0	1	1	0	1	4
FC2	1	1	0	0	1	1	1	0	1	1	7
F4	0	0	0	0	0	1	0	0	1	0	2
F8	1	1	1	0	1	1	1	0	1	1	8
Fp2	0	1	0	1	1	1	0	0	1	0	5
Fz	0	0	0	0	1	0	1	0	1	1	4
Accuracy (%)	81.47	82.69	84.66	84.12	83.70	82.21	68.84	70.53	82.15	83.20	

**Table 3:** Optimal channel selection and recognition accuracy of “No. 14 Couplets”.

Channel	Subject										Total
	2	5	8	11	14	17	20	23	26	29	
Fp1	1	1	1	1	1	1	1	1	1	0	9
F3	0	0	1	1	1	1	1	1	0	0	6
F7	1	0	0	1	1	0	0	0	0	0	3
FT9	1	0	1	1	0	1	1	1	1	1	8
FC5	1	1	1	0	0	0	0	1	1	1	6
FC1	0	1	1	0	0	1	1	0	1	1	6
C3	1	1	1	0	1	1	0	1	1	1	8
T7	1	0	1	0	0	1	0	1	0	1	5
CP5	1	1	0	1	0	1	1	1	0	1	7
CP1	1	0	0	0	0	0	1	0	1	1	4
Pz	1	1	0	1	0	0	0	1	0	0	4
P3	1	1	1	0	1	0	0	1	0	1	6
P7	1	0	0	1	1	1	1	0	0	0	5
O1	1	0	1	1	1	0	0	0	1	1	6
Oz	0	1	0	1	1	1	0	1	1	1	7
O2	1	0	1	0	1	0	1	0	1	1	6
P4	0	1	0	1	1	0	0	1	1	0	5
P8	1	0	1	1	1	1	0	0	1	0	6
CP6	0	1	1	1	0	0	1	1	1	0	6
CP2	1	1	1	0	0	1	1	1	1	0	7
Cz	1	0	1	0	1	1	0	0	0	0	4
C4	0	0	0	1	0	1	0	1	0	0	3
T8	1	1	1	1	1	1	1	1	0	0	8
FT10	0	0	0	1	0	0	0	0	1	0	2
FC6	1	0	0	1	0	1	0	0	0	1	4
FC2	0	0	1	0	0	0	1	0	1	0	3
F4	1	0	1	1	1	0	0	1	0	1	6
F8	0	1	1	0	1	0	1	1	0	0	5
Fp2	0	1	1	0	0	0	1	0	0	0	3
Fz	1	0	1	0	0	0	0	1	1	0	4
Accuracy (%)	81.79	68.57	83.57	82.14	80.36	64.29	82.14	82.50	86.07	74.64	

**Table 4:** Optimal channels selection and recognition result based on “Symphony No. 5 in C minor”.

Channel	Subject										Total
	3	4	9	10	15	16	21	22	27	28	
Fp1	1	1	0	1	1	0	0	1	1	1	7
F3	1	1	0	1	0	1	1	1	0	1	7
F7	1	1	1	1	1	1	0	0	0	1	7
FT9	1	1	1	0	0	1	0	1	0	1	6
FC5	0	0	0	1	0	0	1	0	1	1	4
FC1	1	1	1	1	0	1	0	1	1	1	8
C3	0	0	0	0	1	1	0	0	1	1	4
T7	1	0	0	1	1	1	0	1	0	0	5
CP5	0	1	1	0	1	1	1	1	1	1	8
CP1	1	0	0	0	1	0	1	0	0	0	3
Pz	0	1	1	0	1	1	1	0	1	1	7
P3	1	1	1	1	1	0	0	1	1	0	7
P7	1	1	0	0	0	1	0	0	1	1	5
O1	1	0	1	0	1	1	1	0	1	1	7
Oz	1	1	0	0	0	1	1	1	0	1	6
O2	1	1	1	1	1	1	1	1	0	1	9
P4	1	0	1	1	1	1	0	0	1	0	6
P8	1	1	1	1	0	0	1	1	1	0	7
CP6	0	0	0	1	1	0	1	1	0	1	5
CP2	0	0	1	1	1	1	0	0	1	1	6
Cz	0	0	0	1	0	0	0	1	0	1	3
C4	0	1	1	0	1	0	0	1	1	0	5
T8	1	0	0	1	1	1	0	1	0	1	6
FT10	1	1	1	0	0	1	1	0	0	0	5
FC6	0	0	1	0	0	1	0	1	0	0	3
FC2	0	1	1	1	1	0	0	1	1	1	7
F4	1	0	1	1	1	1	0	0	0	0	5
F8	0	0	1	0	1	0	0	1	1	1	5
Fp2	1	0	0	1	1	0	1	0	1	1	6
Fz	1	0	1	0	1	1	0	1	0	1	6
Accuracy (%)	68.33	72.29	69.38	73.96	76.67	68.33	52.50	49.79	74.17	76.04	

**Table 5:** Two indexes of each channel.

**Channel**	Fp1 *	F3 *	**F7**	**FT9**	**FC5**	**FC1**	**C3**	**T7**	CP5 *	**CP1**
FRSOC	1.265	1.265	0.096	1.145	0.783	1.084	0.783	0.904	1.325	0.783
OCSR (%)	70.0	70.0	53.3	63.3	43.3	60.0	43.3	50.0	73.3	43.3
**Channel**	Pz *	**P3**	**P7**	**O1**	**Oz**	O2 *	**P4**	**P8**	**CP6**	**CP2**
FRSOC	1.265	1.145	1.024	1.145	1.024	1.265	0.904	1.084	0.096	1.145
OCSR (%)	70.0	63.3	56.7	63.3	56.7	70.0	50.0	60.0	53.3	63.3
**Channel**	**Cz**	C4 **	T8 *	FT10 **	FC6 **	**FC2**	**F4**	**F8**	**Fp2**	**Fz**
FRSOC	0.723	0.663	1.325	0.663	0.663	1.024	0.783	1.084	0.843	0.843
OCSR (%)	40.0	36.7	73.3	36.7	36.7	56.7	43.3	60.0	46.7	46.7

* Principal component channel, ** Weak related channel.

## Data Availability

The data and the music experimental materials presented in this study are available on request from the corresponding author.
